# Gender responsive multidisciplinary doctoral training program: the Consortium for Advanced Research Training in Africa (CARTA) experience

**DOI:** 10.1080/16549716.2019.1670002

**Published:** 2019-10-01

**Authors:** Anne M. Khisa, Peter Ngure, Evelyn Gitau, Justus Musasiah, Eunice Kilonzo, Emmanuel Otukpa, Marta Vicente-Crespo, Catherine Kyobutungi, Alex Ezeh, Sharon Fonn

**Affiliations:** a Research Capacity Strengthening, African Population and Health Research Center, Nairobi, Kenya; b Department of Applied and Technical Biology, Technical University of Kenya, Nairobi, Kenya; c Research Capacity Strengthening, African Population and Health Research Center, Nairobi, Kenya; d Department of Community Health and Prevention, Drexel University, Philadelphia & School of Public Health, University of Witwatersrand, Johannesburg, South Africa; e School of Public Health, University of the Witwatersrand, Johannesburg, South Africa

**Keywords:** Gender equality, research capacity building, higher education research, multi-disciplinary doctoral training, Africa, public and population health

## Abstract

Doctoral training has increasingly become the requirement for faculty in institutions of higher learning in Africa. Africa, however, still lacks sufficient capacity to conduct research, with just 1.4% of all published research authored by African researchers. Similarly, women in Sub-Saharan Africa only constitute 30% of the continent’s researchers, and correspondingly publish little research. Challenging these gendered inequities requires a gender responsive doctoral program that caters for women’s gender roles that likely affect their enrollment in, and completion of, doctoral programs. In this article, we describe a public and population health multidisciplinary doctoral training program – CARTA and its approach to supporting women. This has resulted in women’s enrollment in the program equaling men’s and similar throughput rates. CARTA has achieved this by meeting women’s practical needs around childbearing and childrearing and we argue that this has produced some outcomes that challenge gender norms, such as fathers being child minders in support of their wives and creating visible female role models.

## Background

The Consortium for Advanced Research Training in Africa (CARTA) was founded in 2009 by nine universities and four research institutions in Africa in partnership with select northern universities [1]. CARTA aims to promote internationally-competitive research and research training located in African universities. The consortium supports the development of a vibrant African academy able to lead world class multi-disciplinary research that impacts positively on public and population health. To ensure long-term sustainability, a key element of the CARTA program is that the African partner institutions enroll their junior faculty as PhD fellows who register at any of the participating African universities. Fellows’ success and the program’s impact are tracked through various measures that include graduation rates and time to graduation, fellows’ success in winning major research grants, their publications in international peer-reviewed journals, appointments to leadership positions in the academy and beyond, impact of their research outputs in informing national or international policy and practice, and their progression to post-doctoral positions in competitive environments.

In this publication, we seek to examine a key objective of CARTA, from its inception, which is to promote gender equality in higher education and research in Africa. Gender equality has been identified as a contributor to economic efficiency and development [2]. The Global Gender Disparity Gap considers education attainment as one of the four indicators of progress and ranks sub-Saharan Africa (SSA) last on the index [2]. Although the number of women with PhDs in Africa has increased steadily over the last decade, only 30.4% of scientists in Sub-Saharan Africa are women [3]. The gender disparity in science exists against a backdrop of African scientists, in general, contributing proportionally less to global research output. SSA constitutes 1.1% of the world’s scientific researchers, and in 2014, published 1.4% of the world’s total scientific papers [4].

Women’s progress within higher education institutions is circumscribed. Early career growth in academia happens at prime childbearing ages, forcing choice dilemmas for many women. This is compounded by their gender roles. Women not only bear children but their socially constructed roles oblige women to be the primary caregiver and often the sole nurturer limiting women’s pursuit of higher education and doctoral degrees [5].

CARTA recognizes the structural nature of gender relations operating in Africa today where gendered social norms are still prevalent in society at large. Women’s reproductive roles and the expectation of women as home makers and care providers are dominant. This is further reinforced within academia which is hierarchical in nature, expressed as hierarchy between teachers and students, senior professors and junior lecturers and between disciplines [6] as well as between men and women. While CARTA operates a merit-based system in the admission of PhD fellows into the program, it has been mindful of the obstacles women face in taking advantage of PhD training opportunities. For this reason, CARTA instituted a number of interventions that aimed to make CARTA fellowships accessible to, and supportive of, female fellows.

We classify these interventions as addressing women’s practical needs and have located our commentary within the theoretical framework proposed by Molyneux [7] and applied by Moser [8]. Using their framing, strategic gender needs are defined as interventions that overcome women’s subordination and support them to achieve a strategic goal such as women’s gender equality. This is distinguished from women’s practical gender needs that seek to meet women’s immediate needs as a result of their gendered position. CARTA’s interventions address women’s practical needs.

In recruiting PhD fellows, CARTA recognizes that childbearing affects men and women differently; in particular, how candidates are able to combine childbearing with pursuing advanced degrees. CARTA therefore applies a differential maximum age cut-off for female (45 years old) and male (40 years old) applicants to its PhD fellowships. Apart from this differentiation, male and female applicants are assessed purely on merit.

Once fellows are admitted, CARTA enables women to take full advantage of the program’s offerings in ways that are compatible with other gendered roles that would have traditionally limited their participation or progress in a doctoral training program. CARTA PhD fellows can enroll at any one of the African partner universities, including their own institution. They follow the academic rules of the institution in which they register. The CARTA consortium adds value to what is already offered at the various universities by running a set of four interdisciplinary joint advanced seminars (JAS) offered at strategic times during the PhD journey. This exposes fellows to alternative learning and teaching environments. The JASs constitute four one-month long residential programs which bring the fellows in each cohort together with an international faculty teaching a specifically crafted curriculum. The curriculum focus on promoting critical thinking, teaching advanced research skills and preparing fellows for their role as academics; namely to develop independent research programs, train and mentor the next generation, and influence policy and practice through their research. The JASs augment existing training; but particularly challenges institutional hierarchies that are not merit based [6].

This interdisciplinary set of seminars exposes fellows to an internationally competitive curriculum, new ways of thinking, creates a vehicle for them to network with each other across the African institutions, as well as with the international academy who participate in the JAS as facilitators and guest lecturers. The opportunity to register at a fellow’s own university and complementing the university’s doctoral program with the JASs ensures fellows get the required skills and support they need irrespective of whether they chose to study at home or at another African institution. It is this exposure to an alternative learning environment that sets CARTA apart and is one vehicle that is used to ensure internationally competitive graduates. Attendance at each JAS is mandatory.

To ensure CARTA women participants are able to fully participate in the JASs, CARTA supports a breastfeeding mother to attend the JASs with her baby and a child minder who will take care of the baby as the mother participates fully in the month-long residential JAS. CARTA covers the full cost for the fellow, the child, and the child minder. Twenty-three out of the eligible 101 women have taken advantage of the CARTA’s childcare support opportunity so far, with three women using the facility more than two times during their PhD fellowship. As CARTA fellowships admit only faculty members as PhD fellows, it also means that CARTA fellows are full time employees of their institutions and they are entitled to paid maternity leave. Fellows can therefore take advantage of these existing policies. CARTA grants fellows, who request it, a leave of absence during their maternity leave, and restarts their award on their return. They are thus not penalized in any way and eventually enjoy the same benefits as non-pregnant or male fellows.

CARTA offers equal financial support to fellows (stipends and research funds). This is different from the experience many academics have in their usual employment where women academics earn less and control fewer financial resources than their male counterpart’s control.

## Impact

For this paper we report on CARTA fellow’s enrollment and achievements up to December 31^st^, 2018. Of the 199 PhD fellows enrolled in successive cohorts of approximately 25 fellows per year since 2011, 62 fellows had graduated by December 2018. Collectively CARTA fellows have demonstrated success in all the measures described above. This includes publishing 651 articles in peer reviewed journals, noting that some papers are collaborative with more than one fellow on a single paper. In addition, raising more than $9 million USD in research grants in addition to their CARTA awards, being promoted to senior academic positions such as heads of department or assistant deans, and taking up roles on national or international committees.

As shown in Table 1, in the first two years (2011 and 2012) of the program more men than women were admitted into the program. This reversed thereafter and by year eight (2018) 53% of the CARTA fellows were women. Only cohorts 1 to 4 have been in the program long enough to be expected to have graduated. Looking at those four cohorts combined, 67% of CARTA PhD fellows have graduated, a higher percentage of the males (74%) had graduated compared to the females (58%). However, on-time graduation, which is within 4.3 years after enrollment, does not differ for men and women, although only 34% of the total intake, both men and women, graduated on time. This on-time completion rate is relatively impressive given that the fellows continue to teach and supervise students.

**Table 1.** Sex disaggregated data of number of PhD fellows admitted, retained, graduated, graduated on-time, and number of publications produced by each cohort.

	Admitted			Retained PhD fellows^#^			Graduated by December 2018			On-time Graduation^##^			Total Number of Publications by PhD fellows**	No. of fellows who have authored publications**** (Authors output/list)	
Cohort/Year	Total	Male n and %	Female n and %	Total	Male	Female	Total	Male n and % of male fellows retained	Female n and % of female fellows retained	Total	Male n and % of males fellows retained	Female n and % of female fellows retained		Male n(median)	Female n(median)
1/	23	14	9	20	13	7	19	13	6	6	4	2	150	160	26
2011		61%	39%	87%	65%	77%		100%	86%		31%	29%		(8)	(2.5)
2/	21	11	10	18	10	8	13	7	6	7	4	3	114	81	43
2012		52%	48%	86%	91%	80%		70%	75%		40%	38%		(5)	(2)
3/	25	12	13	22	9	13	15	7	8	10	5	5	72	37	40
2013		48%	52%	88%	75%	100%		78%	62%		56%	38%		(4)	(2)
4/	27	15	12	27	15	12	11	8	3	7	4	3	113	89	31
2014		56%	45%	100%	100%	100%		53.%	25%		27%	25%		(5)	(2.5)
***Sub total***				***87***	***47***	***40***	***58***	***35***	***23***	***30***	***17***	***13***			
							***67%***	***74%***	***58%***	***34%***	***36%***	***33%***			
5/	25	12	13	22	10	12	3**	1**	2**	**	**	**	67	38	36
2015		48%	52%	88%	83%	92%								(2)	(4)
6/	25	11	14	24	10	14	1**	0**	1**	**	**	**	76	19	59
2016		44%	56%	96%	90%	100%								(2)	(4)
7	27	8	19	26	7	19	**	**	**	**	**	**	42	5	40
2017		30%	70%											(2)	(2)
8	26	10	16	26	10	16	**	**	**	**	**	**	17	11	7
2018		39%	62%	100%	100%	100%								(1)	(1)
**Total**	**199**	**93**	**106**	**185**	**84**	**101**	**62**	**36**	**26**	**30**	**17**	**13**	**651**	**440**	**282**
		**47%**	**53%**	**93%**	**90%**	**95%**									

**^#^**Some fellows did not take up the offer of a CARTA PhD fellowship or chose to register at a Northern university; CARTA has a contract with PhD fellows which includes a set of deliverables and milestones that must be reached – fellows can be exited from the program for failing to meet these commitments and then are not eligible to graduate as a CARTA fellow. It should be noted that leave of absence can be applied for should unexpected events delay a fellow’s progress, there are no penalties for applying for a leave of absence as long as this is done before a fellow is exited for lack of progress.

**^##^**On-time graduation, assuming each fellow met all their milestones, is 51 months from the first Joint Advanced Seminar during which the monthly stipend to support fellows is first issued.

**These cohorts of PhD fellows had not been in the program long enough for their ‘on time graduation’ to be calculated.

**These are peer reviewed journal articles published by PhD fellows by December 2018. Each publication has been counted only once irrespective of whether more than one fellow is listed as an author.

****No. of fellows who have authored publications is the authorship per fellow and in this case where fellows are joint authors of a publication, we have counted the publication more than once. The total authorship for both male and female, (722) is therefore greater than the total number of publications in each cohort (651). For this analysis, we have excluded publications written after graduation.

**Table 2.** Percentage of male and female CARTA fellows registered at their own (home) or another (away) African university*.

		Male		Female	
Cohort	Total retained Fellows	Home	Away	Home	Away
1	20	62%	39%	71%	29%
2	18	90%	10%	88%	12%
3	22	78%	22%	92%	8%
4	27	73%	27%	50%	50%
5	22	60%	40%	92%	8%
6	24	89%	11%	73%	27%
7	26	71%	29%	63%	37%
8	26	50%	50%	50%	50%
**Total**	**185**	**72%**	**28%**	**72%**	**28%**

*CARTA fellows can register for their PhD at their own institution or any of the African universities that are members of the consortium.

We have taken two approaches to reporting publication output. The first approach is the total number of publications. Here we count each publication only once irrespective of whether more than one fellow is listed as an author. Our second approach is to describe authorship per fellow and in this case where fellows are joint authors of a publication, we have counted the publication more than once. We also note that publication rates can vary across disciplines and the number of publications does not give any indication on the quality of the publications or ranking of the journals. However, this early indication of a potential gender bias in publications rates of fellows that are uniformly trained deserves continued monitoring and deeper assessment so female scholars can be better supported to publish.

CARTA encourages fellows to register at an institution other than their own so that they have an opportunity to experience a culture and, in particular, a learning and research environment different to their own institution. CARTA incentivizes this by offering an additional $250 per month and a return ticket each year between home country and country of study. We expected more male fellows would take advantage of this opportunity as female fellows may feel a greater obligation to remain at their home institution. We analyzed if there was any statistical difference between men and women who registered at their home/other institution and found none. Table 2 shows the registration of male and female fellows in home and host institutions. The similar rate of home registration for both men and women may reflect other imperatives, such as not being able to take time off work to do a PhD fulltime for any staff. It may also be due to loss of certain benefits and reduced salaries when faculty at various African universities go on study leave. CARTA has negotiated reduced teaching loads for all fellows but this may not always be adhered to. However, because the sample of those who have graduated is too small, we cannot yet assess whether registration at home or away has any effect on time to graduation.

CARTA PhD fellow register in various disciplines but all their research topics are relevant to the field of public and population health. Disciplines range from medicine, nursing and midwifery, epidemiology, chemistry, statistics, nutrition, anthropology, geography, linguistics, sociology, veterinary medicine, zoology, environmental sciences and engineering. There is a good mix by gender within most disciplines; we have both male and female chemists, male and female statisticians and we even admitted both a male and female engineer. However, while the within-discipline sample size was too small to do any formal testing, we note that nursing and midwifery showed an obvious predominance of women.

## Discussion

The simple approaches CARTA has taken to support female fellows: i.e. differential age cut-off for admission and support for mothers of young children, respond to obvious practical needs female doctoral students in Africa may face. It has proved effective in attracting female candidates to the program even when selection is purely based on merit (53% of the fellows are women). Since the third cohort, and as information about CARTA’s commitment to women academics became better known, more women have continued to apply for the fellowship and they have been as competitive as their male counterparts.

The time taken to complete a PhD in sub-Saharan Africa can be unusually long and throughput in doctoral programs can be quite low. CARTA’s support to female fellows has shown that female doctoral fellows can achieve the same rate of on-time completion of their training as their male counterparts. In general, both female and male CARTA fellows have taken on average 4 years and two months to complete their degrees. However, overall (compared to on-time) completion rate is higher for men compared to women. This may reflect women’s reproductive and domestic roles and responsibilities, which may interfere with the time they can devote to their studies. It is also the case that some kinds of research, for instance longitudinal studies, take longer. We have not explored the role this factor played in the observed differences in overall completion rates between male and female CARTA fellows.

Nonetheless, CARTA’s average time to completion (about 4.3 years) is comparable to, or better than, rates observed elsewhere outside Africa: in the Netherlands both male and female candidates took 4.9 years [9]; 5.9 years in Canada [10]; and even longer times have been reported for the USA [11]. We ascribe the relatively good completion time to CARTA’s structured approach to PhD training, in particular the JASs and the various milestones that fellows have to meet.

The differential output of publications where men seem to be producing more publications compared to women requires further investigation and is a current area of research we are undertaking. Taking time to publish is often done outside working hours and it may be that men have more control over their time than women. As publication output is often used as a promotion criterion, this could limit women’s advancement in academia if not addressed.

Seventy-two percent of CARTA fellows are registered in their home institutions. Being able to register and stay at home to complete a PhD was something that women in particular stated that they valued and it allowed them to take up this scholarship as opposed to those that require registration and time at a northern university [12]. As reported in an external evaluation of CARTA commissioned by Sida: ‘Female fellows also report that the overall CARTA model, where they do not need to leave their families for extended periods of time, is far more appropriate for their needs than PhD fellowships in the North, and that they might not have been able to pursue a doctorate in the North for these reasons. One female fellow said that without the support from CARTA’s policies, she “would probably have dropped out”’ [12]. This risk of studying at the institution where one is employed, however, is that it limits the individuals’ networking, exposure to new ideas, people, disciplines and practices. The way CARTA is structured: allowing registration across African institutions; adopting a cross-institutional, multidisciplinary cohort approach; and instituting the JASs with its international faculty and alternative pedagogic approach, has enabled CARTA to provide a gender-sensitive program without losing the benefit of exposure to alternative internationally competitive learning environments. We had anticipated that more men would register at a university other than their own but this has not been the case, especially with more recent cohorts of fellows.

There is evidence that the environment in which women academics work appears to value them less as illustrated by the differential pay between men and women. This gender pay-gap in academia is an almost universal phenomenon and requires concerted action of a strategic type internationally [13,14]. We also note that even if our fellows have been afforded a more gender sensitive learning environment, they may still be limited in their progress if they return to a less gender sensitive institution. We recognise the need to address women’s strategic needs; the CARTA program has done this in the way it models leadership, challenges gratuitous hierarchy, and in its attempt to influence university systems. However strategic change is a long-term endeavour that requires a range of strategic alliances using multiple methods. For instance, a gender responsive university environment in Australia relies on gender equality measures at national level such as frameworks, commitments and actions with setting and monitoring of targets and robust data collection [15]. At university level, setting key performance indicators for the university, transparent processes for recruitment and promotion, and support networks for women have provided necessary infrastructure to ensure a gender responsive university environment [15]. CARTA partner universities may consider such structural adjustments to provide a conducive environment for women to succeed.

In this paper, we focus only on how a capacity-building programme can address practical needs. Nonetheless, we argue that the practical nature of the CARTA interventions may have some strategic value. Supporting breastfeeding mothers to attend JASs has allowed women to pursue their PhD and motherhood simultaneously. The first CARTA fellow to use this childcare provision in 2011 brought her husband as the care-giver for the entire four-week JAS. Every four hours he would arrive at the training venue with their 4-month-old baby who was passed along the row to her mother to breastfeed. The husband noted this was a unique opportunity to actively parent as a man, something he would not have done were it not for CARTA’s approach and would not have done had he been at home with his wife and child. It demonstrated a subversion of gender norms for all participants. This happened again in 2019 with the father expressing similar sentiments. However, in general, most fellows brought either a formally employed child minder, sister, or mother with them. The external evaluation of CARTA described this practice as not only creating greater opportunities for women to benefit from CARTA, but also a symbol of what a genuine commitment to gender equality means in practice [12]. CARTA has given practical expression to assertions that women should be allowed to travel with their children and that such travel should be paid for by the program as has been suggested by others [16]. These interventions, however, have not been able to address all disparities among our female and male fellows. Male fellows have higher overall graduation rate and they are publishing their research at a higher rate than female fellows. CARTA remains committed to understanding practical ways the program can support female doctoral students and assisting them to succeed in academia after obtaining their PhDs.

There are other examples of how we believe CARTA’s practical interventions have strategic impact. For instance, one aspect of CARTA fellows’ productivity is demonstrated in their ability to attract and win internationally competitive research grants. In the reporting period of 2017 and 2018, fellows raised a total of 3,666,475 USD. Notably, women fellows raised 40.4% of these funds, a number of which are major research grants. These successful women provide role models for other women researchers in academia.

A number of CARTA women graduates have gone on to leadership roles, earned promotions and serve on committees. This includes four women graduates who have been promoted and now occupy positions as deans of faculty and/or heads of department at the Universities of Ibadan, Malawi and the University of the Witwatersrand. The promotions have changed the gender composition of decision makers in their institutions. While it is not a guarantee that this will improve gender equity for others, it does begin to change the status quo. Many of these graduates link their promotion to the experience they have had through CARTA. While we cannot directly attribute the promotions solely to CARTA, the fellows themselves and anecdotal evidence suggest that their fellowships have played a key part in their promotions and successes.

Women in the CARTA program have had their practical needs met during training and this has supported them to maintain on-time graduation rates that are similar to their male counterparts. In addition, some of the female fellows have advanced in the academy, reaching positions of power and in decision-making. Whilst the CARTA program has managed to address practical gender needs, structural barriers like unequal pay and unfair workloads can be addressed by working with institutions to change their policies.

With higher education institutions increasingly requiring a PhD qualification for faculty, changing gender power relations should begin at doctoral training programs, ensuring parity and sustained participation of men and women in university faculty career trajectories. CARTA is addressing inequities that bar women’s progress in knowledge production by addressing their practical needs in doctoral training. More importantly, CARTA’s JASs are also used to reinforce the importance of gender equality in socioeconomic development and to challenge traditional norms and values that support or sustain gender inequality, including how these are manifested in higher education systems in Africa. CARTA has done this without compromising on merit. We have also illustrated that meeting women’s practical needs is possible and suggest that this can be applied to many programs beyond our own. This view is supported by an external independent evaluation of CARTA commissioned by Swedish International Development Agency (Sida) in 2015. The report found that CARTA was an example of a successful model in both supporting women researchers and in promoting an appreciation of gender as a determinant of health and development [12].

## Conclusion

The CARTA program is helping to narrow gender gaps in admissions to PhD training, in winning competitive research grants, and also rising to academic leadership positions. CARTA will evaluate the reasons behind the apparent lower publication rate of women to assess how this too may be overcome. Although CARTA has not reversed SSA regional gender gaps, it is contributing to reducing them.

## Paper context

Enrolling and succeeding in a PhD program is influenced by various factors, including gender. Consequently, few women researchers in Sub-Saharan Africa contribute to publications and hold academic positions of leadership. We enhance knowledge on how CARTA doctoral program has responded to the needs of students arising from their gendered roles, in SSA context, to contribute to narrowing gender gaps in admission to PhD, winning research grants, publishing and rising to academic leadership positions.
